# Characterisation of stemness and multipotency of ovine muscle‐derived stem cells from various muscle sources

**DOI:** 10.1111/joa.13420

**Published:** 2021-02-27

**Authors:** Mohamed I. Elashry, Kateryna Gaertner, Michele C. Klymiuk, Asmaa Eldaey, Sabine Wenisch, Stefan Arnhold

**Affiliations:** ^1^ Institute of Veterinary Anatomy, Histology and Embryology Justus‐Liebig‐University of Giessen Giessen Germany; ^2^ Clinic of Small Animals c/o Institute of Veterinary Anatomy, Histology and Embryology Justus‐Liebig‐University of Giessen Giessen Germany; ^3^ Anatomy and Embryology Department Faculty of Veterinary Medicine University of Mansoura Mansoura Egypt

**Keywords:** cell differentiation, multipotency, skeletal muscle, stem cells

## Abstract

Muscle stem cells (MSCs) are a promising tool for cell‐based therapy and tissue regeneration in veterinary medicine. Evaluation of MSCs from muscles of different origins improves our understanding of their regenerative potential. The present study compared the stemness, cell proliferation, migration potential, myogenic differentiation (MD), and multipotency of MSCs for four developmentally different muscles of ovine origin. MSCs were isolated from the hind limb (HL), diaphragm (DI), extraocular (EO), and masseter (MS) muscles. Cell proliferation, migration, and stemness were examined using sulforhodamine B, and colony formation assays. Evaluation of multipotency was examined using histological and morphometric analyses, alkaline phosphatase (ALP) activity, and the expression of myogenic, adipogenic, and osteogenic markers using RT‐qPCR. Data were statistically analysed using analysis of variance. The results revealed that all experimental groups expressed stem cell markers paired box transcription factor Pax7, α7‐integrin, CD90, and platelet‐derived growth factor receptor alpha. DI and HL muscle cells displayed higher proliferation, migration, and colony formation capacities compared to the EO and MS muscle cells. HL and DI muscle cells showed increased MD, as indicated by myotube formation and relative expression of *MyoD* at day 7 and *Myogenin* at day 14. Although MS and EO muscle cells displayed impaired MD, these cells were more prone to adipogenic differentiation, as indicated by Oil Red O staining and upregulated fatty acid‐binding protein 4 and peroxisome proliferator‐activated receptor gamma expression. DI muscle cells demonstrated a higher osteogenic differentiation capability, as shown by the upregulation of *osteopontin* expression and an elevated ALP activity. Our data indicate that ovine HL and DI MSCs have a higher regenerative and multipotent potential than the EO and MS muscle cells. These results could be valuable for regional muscle biopsies and cell‐based therapies.

## Graphical abstract

The data revealed that the cells of the trunk mesoderm possess a greater regenerative potential compared to those cells of head mesoderm. Head muscle cells had a higher adipogenic capacity compared to the trunk muscle cells. In contrast, cells of trunk muscles displayed enhanced osteogenic potential. Collectively, these results suggest that muscle stem cells derived from the paraxial mesoderm and head mesoderm followed a different myogenic programme. Additionally, we could assume that the trunk muscle cells are more suitable sources for biopsies and stem cell isolation for tissue regeneration.

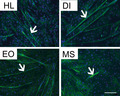

## 1 INTRODUCTION

Skeletal muscle stem cells (MSCs) are a valuable tool for investigating myogenic differentiation (MD) and muscle regeneration. It is well known that stem cells and muscles develop concomitantly. During embryonic development, a group of cells express paired box transcription factor 3 (Pax3), delaminate from the dermomyotome, and migrate to the mesenchyme of the limb bud to differentiate into limb muscles (He et al., 2003; Zammit et al., 2004). Although the limb bud cells expressing Pax3/Pax7 undergo proliferation and differentiation, another group of cells withdraws from the cell cycle to become MSCs (Zammit et al., 2004). In 1961, adult MSCs were identified between the basal lamina and sarcolemma of muscle fibres (Mauro, 1961). Adult quiescent MSCs express the paired box transcription factor Pax7 (Shioi et al., 1995), which is essential for their specification and survival (Kuang et al., 2006). In contrast, Pax3 is expressed only in a few muscle groups, including the diaphragm (DI) (Redshaw & Loughna, 2012). Unlike the limb and trunk muscles, head muscles, including the masseter (MS) and extraocular (EO) muscles, develop from the cranial paraxial mesoderm (Harel et al., 2009). In addition, the progenitor cells of the head muscles, excluding the tongue, do not express Pax3 (Zammit et al., 2004).

Adult skeletal muscles show remarkable regenerative potential under physiological (Parise et al., 2008) and pathological conditions (Lepper et al., 2011). Upon muscle injury, MSCs undergo both asymmetric and symmetric cell division (Kuang et al., 2007). In asymmetric cell division, Pax7^+^/myogenic regulatory factor 5 (Myf5^–^) parental cells give rise to two dissimilar daughter stem cells: one is a Pax7^+^/Myf5^–^ cell, whereas the other is a Pax7^+^/Myf5^+^ cell committed to MD. Proliferating MSCs express *MyoD* and are referred to as myoblasts. MD is accompanied by downregulation of *Pax7* and upregulation of *Myogenin* expression, which, together with *MyoD*, is able to activate muscle‐specific contractile protein production (Zammit et al., 2004, 2006).

MSC multipotency has been previously reported in cells with intact muscle fibres that give rise to adipocytes and osteocytes (Asakura et al., 2001; Elashry et al., 2017). MSCs differ in their multipotency. In this context, it has been reported that CD56^+^/CD34^–^ cells isolated from porcine skeletal muscle display myogenic potency, whereas CD56^+^/CD34^+^ cells show both adipogenic differentiation (AD) and MD capacities (Perruchot et al., 2013). Similarly, CD56^+^/CD44^+^/CD45^–^ MSCs were able to differentiate into myotubes, adipocytes, and osteocytes in human beings (Coppi et al., 2006). Furthermore, studies have also revealed that only platelet‐derived growth factor receptor alpha (PDGFRα)‐positive cells showed efficient AD *in vitro* and *in vivo* (Oishi et al., 2013; Watt et al., 1987). Characterising fibro‐adipogenic progenitors as interstitial cells yielded similar marker expression (PDGFRα^+^/CD34^+^/stem cell antigen‐1 (Sca‐1^+^)/CD45^–^/α7‐integrin), but gave rise only to adipogenic and fibrogenic lineages. Although fibro‐adipogenic progenitors cannot develop into myofibers, these cells act as a source of signals that enhance the differentiation of myogenic progenitors in co‐culture experiments (Joe et al., 2010).

The limited use of MSCs in regenerative medicine, owing to their poor survival rate and cell population heterogeneity, necessitates the search for alternative sources. Although several studies have characterised MSCs isolated from the paraxial mesoderm, less attention has been given to MSCs derived from the head mesoderm. Ovine MSCs are very similar to human beings MSCs in terms of their life span, metabolism, proliferation, and differentiation capacity (Yan et al., 2013). Moreover, ovine skeletal muscle contains similar myosin heavy chain isoforms as human beings, including type I, IIA, and IIX (Andruchov et al., 2004; Hemmings et al., 2009; Lefaucheur et al., 1998; Wieczorek et al., 1985). Although it has been reported that MSCs of ovine limb muscles can undergo MD, AD, and osteogenic differentiation (OD) (Yan et al., 2013), ovine MSC populations derived from developmentally different muscle groups have not been fully elucidated. Thus, the present study characterised the stemness and multipotency of MSCs isolated from developmentally different sources of ovine skeletal muscles. We hypothesised that MSCs derived from the paraxial mesoderm would display greater regenerative potency and multipotent properties than those derived from the head mesoderm, including the MS and EO muscles. To that end, we examined MSC stemness, cell viability, cell proliferation, and migration capability using 3‐(4,5‐dimethylthiazol‐2‐yl)‐2,5‐diphenyltetrazolium bromide (MTT), Sulforhodamine B (SRB), wound healing, and colony‐forming unit ability (CFU) assays. MD of various cell populations, as well as their multipotency regarding the adipogenic and osteogenic fate, were examined using immunohistochemistry, morphometric measurements, and RT‐qPCR. The data demonstrate the effect of muscle development on the properties of their own stem cells. We show that MSCs of hind limb (HL) and DI muscles have enhanced proliferation, colony formation, and migration potential in comparison to MS and EO muscle cells. We report that cells of the trunk mesoderm, including HL and DI, demonstrate enhanced myogenic potential compared to the cells of the head mesoderm. Although MS cells have the properties of impaired stem cells, these cells display a higher adipogenic potency. These results are important considering regional muscle biopsies for cell‐based therapy and tissue engineering.

## 2 MATERIALS AND METHODS

### 2.1 MSC isolation and expansion

MSCs were obtained from 4‐week‐old male lambs (*n* = 6). MSCs were isolated from the gastrocnemius of the HL and DI, MS, and EO muscles. Muscle tissue was obtained from the Institute of Veterinary Pathology, Justus‐Liebig University, Giessen, following standard and ethical regulations. Briefly, 1 mm^2^ muscle pieces were transferred in cold phosphate‐buffered saline (PBS, Gibco) with 1% penicillin/streptomycin (P/S, AppliChem). On a clean bench, fat, connective tissue, and blood vessel fragments were removed, and then, the muscle tissue was cut into small fragments using a sterile scalpel blade, placed into 50 ml falcon tubes with PBS, and centrifuged at 670 *g* for 5 min. The supernatant was discarded, and the pellets were digested with 0.2% collagenase IV diluted in 4.5 g/L Dulbecco's Modified Eagle Medium (DMEM, Gibco) on a shaker at 37°C for 40 min. Muscle samples were triturated mechanically using an 18‐gauge needle to obtain single‐cell suspension. An equal volume of 2% foetal calf serum (FCS, Biocell) in DMEM was added, and the mixtures were filtered through a 70‐μm cell strainer, followed by centrifugation at 240 ***g*** for 5 min. Cell pellets were mixed with 1 ml of growth medium consisting of DMEM, 10% FCS, and 1% P/S. To select stem cell populations from other cell types, we used several validation criteria, including pre‐plating, PCR, immunohistochemistry, and flow cytometry, to track stem cell and myogenic marker expression. Briefly, the cells were pre‐plated in 20% FCS in Ham's F10 medium (Gibco) with 1% P/S in plastic culture dishes for 48 h. This method not only preserves the myogenic population in favour of other cell types, but also removes the fast adherent cells, particularly fibroblasts, as previously reported (Chirieleison et al., 2012; Elashry et al., 2017). Non‐adherent cells were cultivated in a fresh medium for expansion. In parallel, cells from each group were examined for stem cell markers, including *CD90*, *MyoD*, *Myogenin*, and *PDGFRα*, using PCR, as well as immunohistochemistry for Pax7, Myogenin, CD44, CD90, and α7‐integrin. Cell populations showing less than 80% positivity for stem cell markers were discarded.

### 2.2 Flow cytometry analysis

Briefly, 1 × 10^6^ cells/ml of fresh medium was used. A volume of 100 μl of cell suspension per well was transferred to a 96‐round‐bottomed‐well plate. After centrifugation at 400 ***g*** for 3 min at room temperature (RT, 15–25°C), cell pellets were washed in a buffer, comprising 1% bovine serum albumin, 0.01% NaN_3_, 0.5% goat serum, and 10% horse serum in PBS, and centrifuged at 400 ***g*** for 3 min at RT. The pellets were incubated with 50 μl of the primary antibodies, including mouse CD44 (1:100 antibody‐online ABIN94121), CD45 (1:100, antibody‐online ABIN319753), and major histocompatibility class II (MHCII, 1:200, Bio‐Rad), for 20 min at RT and then centrifuged at 400 *g* for 3 min. The supernatant was discarded, and the pellets were washed twice in the washing buffer for 3 min, followed by incubation with 50 μl of anti‐mouse‐PE (1:200, BD Biosciences) secondary antibody for 20 min in the dark. After two washing steps, the pellets were suspended in PBS for flow cytometry analysis using an Accuri C6^®^ (BD Biosciences) equipped with Accuri C6 software (BD Biosciences).

Selected cell populations were expanded in the growth medium in T‐75 culture flasks at 37°C and 5% CO_2_. The cells were detached using trypsin (TrypLE, Gibco) at 37°C and 5% CO_2_ for 6–7 min, and then centrifuged at 240 *g* for 5 min. Cell pellets were resuspended in fresh medium and counted. Subsequently, the cells were either passaged or cryopreserved for further experiments. For cryopreservation, 1 × 10^6^ cells/ml were resuspended in freezing medium, containing DMEM, 1% P/S, 30% FCS, and 5% dimethyl sulfoxide (DMSO, Roth), and stored in cryotubes at −160°C for further experiments. Cells from passages P1–P4 were used for all experiments.

### 2.3 MTT assay

The MTT assay (Sigma‐Aldrich) was used to assess the degree of tetrazolium reduction by nicotinamide adenine dinucleotide phosphate (NADPH)‐dependent cellular oxidoreductase, indicating cell viability. Cells of the HL, DI, MS, and EO muscles were seeded into 24‐well plates at a seeding density of 5 × 10^3^ cells/well for 3, 5, and 7 days. Briefly, the medium was replaced with 300 μl of growth medium, having 5 mg/ml MTT diluted in PBS, per well. The cells were incubated for 3 h at 37°C and 5% CO_2_. The solution was removed, and the cells from each muscle group was incubated with 200 μl of DMSO per well in triplicate. The plates were incubated in a shaker for 10 min at RT. Samples (200 μl) for each condition were pipetted in triplicate into a 96‐well plate. Absorbance was measured at 570 nm using a microplate reader equipped with Magellan^TM^ Data Analysis Software (Tecan).

### 2.4 SRB assay

The SRB assay measures the amount of cellular protein indicative of cell number, as previously reported by Vichai and Kirtikara (2006). MSCs were seeded in 24‐well plates at a density of 5 × 10^3^ cells/well in growth medium for 3, 5, and 7 days. Briefly, following the cultivation time points, cells were fixed in 4% paraformaldehyde (PFA, Roth) for 10 min. After three washes in PBS for 5 min each, the cells were incubated with 500 μl of SRB solution, containing 0.4% SRB sodium salt (Sigma‐Aldrich) diluted in 1% acetic acid (Merck) for 10 min at RT. The SRB solution was removed, and the cells were washed with 1% acetic acid five times for 5 min. The bound SRB dye was dissolved in 500 μl/well of 10 mM non‐buffered 2‐amino‐2‐(hydroxymethyl)‐1,3‐propanediol (TRIS, Sigma‐Aldrich) solution (pH 10) for 30 min at RT. A volume of 200 μl from the mixture was pipetted in triplicate per muscle for each animal (*n* = 6) into 96‐well plates. Absorbance was measured at 565 nm using a microplate reader (Tecan).

### 2.5 Wound‐healing assay

Sterile silicon inserts were tightly placed in the middle of each well (1 insert/well) in a 24‐well plate. MSCs of the HL, DI, MS, and EO muscles were seeded in growth medium at a density of 5 × 10^3^ cells/well in triplicate. MSCs were cultivated at 37°C and 5% CO_2_ until they reached 80% confluence. After 24 h, the inserts were carefully removed and fresh medium was added. The cells were examined under an inverted microscope (Axioobserver, Zeiss), and images were captured for up to 48 h. At the removal of culture inserts, the time was recorded as zero (0). Images were taken at 10× magnification using a Leica MC170 microscope (Leica Microsystems) equipped with Leica Application suite (LASV4.4) imaging software (Switzerland). MSC migration to close the intercellular gap was measured after 6, 12, 24, and 48 h. Image analysis and measurements of gap closure were performed using Adobe Photoshop CS6.

### 2.6 CFU assay

MSCs were seeded at a density of 1 × 10^2^, 5 × 10^2^, and 1 × 10^3^ cells/25 cm^2^ in culture flasks with growth medium for 7 days. The medium was changed every 2 days. The cells were washed in PBS and fixed in 4% PFA for 10 min. Crystal violet (5 mg/ml, Roth), diluted in 2% ethanol, was added to each flask for 8 min. The extra stain was washed with distilled water three times for 3 min each. The flasks were left to dry overnight at RT. Cell colonies stained in violet were identified and photographed using a Leica Microscope. The total number of colonies were counted per flask at 4× objective. Then, each flask was divided into four equal squares, and the colonies inside each square were counted. The cell colonies were categorised into three groups: small (10–20 cells), medium (21–50 cells), and large (>50 cells).

### 2.7 Induction of MD

The cells were seeded into 24‐well plates at a density of 1 × 10^4^ cells/well in growth medium. Upon reaching 70% confluency, the growth medium was switched to MD medium containing DMEM, 2% horse serum (Biocell), 20 ng/ml fibroblast growth factor (Thermo Fisher Scientific), and 1% P/S for up to 14 days.

### 2.8 Induction of AD and OD

To examine multipotency, MSCs were induced into adipogenic and osteogenic fates using a specific induction medium. MSCs were cultivated in a growth medium for 48 h. Next, the medium was removed and the cells were incubated in AD and OD media for 14 and 21 days, respectively. The AD medium comprised 4.5 g/L glucose DMEM, 5% FCS, 1% P/S, 0.1 µM dexamethasone (Sigma‐Aldrich), 5 µg/ml insulin‐transferrin‐selenium, and 5 µM rosiglitazone (Sigma‐Aldrich). The OD medium consisted of 1 g/L glucose DMEM, 5% FCS, 1% P/S, 1 mM dexamethasone, 1 M β‐glycerol phosphate (Sigma‐Aldrich), and 100 mM ascorbic acid 2‐phosphate (Sigma‐Aldrich). Cells from each muscle were grown in parallel in basal medium (BM) containing DMEM supplemented with 5% FCS and 1% P/S, which served as non‐induced negative controls.

### 2.9 Alkaline phosphatase activity assay

Cells were incubated with 1% Triton X‐100 (pH 7.4, Calbiochem) for 10 min at RT on days 7, 14, and 21 after osteogenic induction. Cells were detached using a cell scraper, and the lysates were centrifuged at 28,400 × *g* for 2 min. Lysates were incubated with para‐nitrophenylphosphate (NPP, 2 mg/ml, Roth) substrate, dissolved in a buffer solution, containing 1 M TRIS and 5 mM MgCl_2_ (pH 9.0), in triplicate at 37°C for 2 h. The mixture was transferred into 96‐well plates, and the ability of alkaline phosphatase (ALP) to metabolise NPP into p‐nitrophenol (PNP) was measured as previously reported (Bessey et al., 1946). The absorbance of PNP was measured at 405 nm using a microplate reader (Tecan) and Magellan^TM^ Data Analysis Software.

### 2.10 RT‐qPCR

Throughout the study, RNA samples were isolated from primary MSCs and from cells induced to MD and AD at days 7 and 14, and OD at days 7, 14, and 21. Non‐induced cells in the BM were processed in parallel, and served as negative controls for all experimental setups. Approximately 1 µg RNA samples were extracted from all the groups using an RNA purification kit (Sigma‐Aldrich). RNA samples were treated with a recombinant DNAse I (Roche) and RNase inhibitor (Thermo Fisher Scientific), and then reverse‐transcribed into cDNA using a Multiscribe^TM^ Reverse Transcriptase (Thermo Fisher Scientific). Parallel preparation without reverse transcriptase was used as the negative control. The following cycling conditions were applied: 21°C for 8 min, 42°C for 15 min, 99°C for 5 min, and 5°C for 5 min, followed by cooling to 4°C. The obtained cDNA was mixed with a master mix containing Biotherm Taq Polymerase, ultrapure water, buffer, dNTP, and the respective forward and reverse primers. Random hexamers (Microsynth AG) were used as primers as listed in Table 1. PCR was performed for 35 cycles using the following cycling conditions: 5 min at 95°C, 30 s at 94°C, 30 s at 60°C, 30 s at 72°C, and 1 min at 72°C. Gel electrophoresis was performed to evaluate the PCR products. Relative expression of MD (*MyoD* and *Myogenin*), AD (peroxisome proliferator activated receptor gamma, *PPARγ* and fatty acid‐binding protein, *FABP4*), and OD (osteopontin, *OPN*) markers was evaluated in triplicate via RT‐qPCR using the GoTaq qPCR Mix (Promega). A thermal cycler was used for 2 min at 95°C, 15 s at 95°C, 30 s at 60°C, 5 s at 60°C, and 5 s at 95°C for 40 cycles using Bio‐Rad CFX Manager 2.1 software (Bio‐Rad Laboratories GmbH). *18S1* was used as an endogenous reference, and the relative gene expression was quantified using the 2‐^∆∆CT^ method, as described previously (Schmittgen & Livak, 2008).

**TABLE 1:** Primers sequences used for PCR analysis

Gene	Forward	Reverse	Size (bp)
18S.1	ATGCGGCGGCGTTATTCC	GCTATCAATCTGTCAATCCTGTCC	204
Pax7	CTCCGACCTGTGCTGTATTC	GTCATCCGTCACCCTTGAAG	120
MyoD	GTCAACGAGGCCTTCGAGAC	GCGCCTGCAGGCCTTCGATA	114
Myogenin	AAGACAAGGGGCTGGGGCC	TCTTGAGTCTGCGCTTCTCC	134
PDGFRα	TGGAGGACGAAGATTCAGTC	GCCTTGTCTGCTGTCATAGG	112
CD90	CCCGTGGGCAGAAGGTGAC	TCAGGCTGAACTCATACTGGATGG	114
OPN	TGAAACCCCTGATGACTCTGAC	TGTGCTTTCCGTAGGGAAAGG	126
FABP4	ATCAGTGTAAATGGGGATGTG	GACTTTCCTGTCATCTGGAGTGA	117
PPARγ	CACTATGGAGTTCATGCTTGTG	CGGCAGTACTGGCATTTATTTCT	113

### 2.11 Phalloidin staining

After day 7 of MD, the cells were fixed in 4% PFA, washed with PBS twice for 3 min, and incubated with 2.5% phalloidin (Sigma‐Aldrich) diluted in PBS in the dark for 30 min at RT. Then, the cells were washed three times for 3 min with PBS, and their nuclei were counterstained using 4′,6‐diamidino‐2‐phenylindole (DAPI, Thermo Fisher Scientific). The cells were washed twice in PBS for 3 min and photographed under an Axio‐imager fluorescence microscope equipped with a digital camera (Zeiss). Evidence of MD via the formation of myotubes was assessed at 10× magnification. Myotube length (*n* = 20), width (*n* = 20), number of nuclei per myotube (*n* = 17), volume of cytoplasm (*n* = 20), and number of myotubes per microscope field (*n* = 10) per muscle were recorded using an AxioVision Microscope Software (Zeiss).

### 2.12 Oil Red O staining

The cells were fixed in 4% PFA at days 7 and 14 post‐AD, and then washed twice in distilled water for 3 min. Briefly, the cells were washed with 60% isopropanol for 5 min, and incubated with freshly prepared Oil Red O (Sigma‐Aldrich) staining solution diluted 2:3 in distilled water for 30 min at RT. The fat droplets were recognised in red under an inverted light microscope. Non‐induced cells were processed in parallel and served as negative controls. For semi‐quantification of Oil Red O staining, 200 μl of 100% isopropanol was added in triplicate into each experimental group for 30 min at RT with shaking. The staining extracts were transferred in triplicate to a 96‐well plate. The absorbance was measured at 492 nm using a Tecan microplate reader equipped with Magellan^TM^ Data Analysis Software.

### 2.13 Alizarin Red S staining

The cells previously induced for OD were fixed in 4% PFA at days 7, 14, and 21 post‐induction. The cells were washed twice for 3 min with distilled water, and then incubated with 2% Alizarin Red S (Roth) in distilled water for 15 min at RT. After three washing steps in distilled water, the matrix mineralised by calcium deposits was recognised as having a red‐orange colour, and was photographed under an inverted light microscope.

### 2.14 Immunocytochemistry

MSCs were seeded on sterile glass coverslips in 24‐well plates at a seeding density of 1 × 10^4^ cells/well. The cells were fixed in 4% PFA (pH 7.4) for 10 min at RT. Then, the cells were washed with PBS three times for 3 min. The cells were permeabilised with 0.5% Triton‐X100 diluted in PBS for 10 min at RT. Thereafter, the cells were washed in PBS three times for 5 min and blocked in a buffer containing 0.1% Tween and 5% goat serum diluted in PBS for 30 min at RT. The cells were then incubated with primary antibodies, including anti‐mouse Pax7 and anti‐mouse Myogenin (1:30, DSHB, University of Iowa), α7‐integrin (1:50, Santa Cruz Biotechnology), anti‐mouse MyoD (1:100, BD Biosciences), and anti‐mouse CD90 (1:50, Antibody‐online GmbH), diluted in blocking buffer at 4°C overnight. The primary antibody was detected using Cy3 goat anti‐mouse secondary antibody (Dianova), diluted 1:100 in blocking buffer after incubation for 1 h in the dark at RT. The nuclei were counterstained with DAPI (Thermo Fisher Scientific) diluted in blocking buffer 1:5000 after incubation for 5 min in the dark at RT. To detect Ki67 nuclear antigen, cells were incubated overnight with rat monoclonal anti‐Ki67 primary antibody (1:200, diluted in the blocking buffer, Dako) at 4°C. The latter was detected using a mouse polyclonal horseradish peroxidase‐conjugated secondary antibody (HRP, 1:500, Dianova) after incubation for 30 min at 37°C. HRP was visualised by providing a substrate composed of 4 mg/ml dimethylformamide 3‐amino‐9‐ethylcarbazole and 2.5 µl H_2_O_2_ diluted in 50 mM sodium acetate (pH 5.2) for 30 min. The coverslips were mounted on microscope slides using Dabco‐Mowiol (Roth), and the cells were photographed using a fluorescence microscope equipped with AxioVision Microscope Software. Quantification of Pax7 in HL, DI, EO, and MS muscle cells was performed using the counting tool provided by Axiovision Microscope Software. Pax7 positive cells were counted in a minimum of 10 random microscopic fields all over the cover slip. For all experimental groups, cells processed without adding primary antibodies served as negative controls (Figures S1e, j, o, t, y, and S2e,j).

### 2.15 Statistical analysis

The data were collected from MTT, SRB, CFU, and wound‐healing assays, as well as ALP activity and Oil Red O semi‐quantification in triplicate (*n* = 6 per experimental group). Two‐way ANOVA was performed to evaluate the effect of cell source (variable 1), i.e. HL, DI, MS, and EO muscles, and the time required for the gap closure (variable 2), indicative of efficient regeneration. Two‐way ANOVA was also carried out to analyse the effect of cell source (variable 1) at different time points (variable 2), including 3, 5, and 7 days, on both cell viability and cell protein content. The effect of muscle source and seeding densities (100, 500, and 1000 cells/flask) on the colony formation ability was also assessed using two‐way ANOVA. Non‐parametric one‐way ANOVA was performed to evaluate the number of Pax7‐positive cells per microscopic field (*n* = 10) for all muscle groups. Effect of MD on the number, width, and length of myotubes; number of myonuclei per tube; and nuclear cytoplasmic ratio of the myotubes for all muscle groups was also examined using one‐way ANOVA. To analyse the effect of differentiation medium (variable 1), including AD and OD versus BM on ALP activity, Oil Red O semi‐quantification, and the quantification of relative expression of markers at various time points (variable 2), two‐way ANOVA was performed. Multiple comparisons were performed and variable interactions were assessed using Tukey's and Sidak's post‐hoc tests. The output is presented as mean ± SEM. Statistical significance was set at *p* < 0.05. Statistical analyses were performed using GraphPad Prism 7.0 software.

## 3 RESULTS

### 3.1 Evaluation of proliferation and migration of MSCs from various sources

**FIGURE 1:**
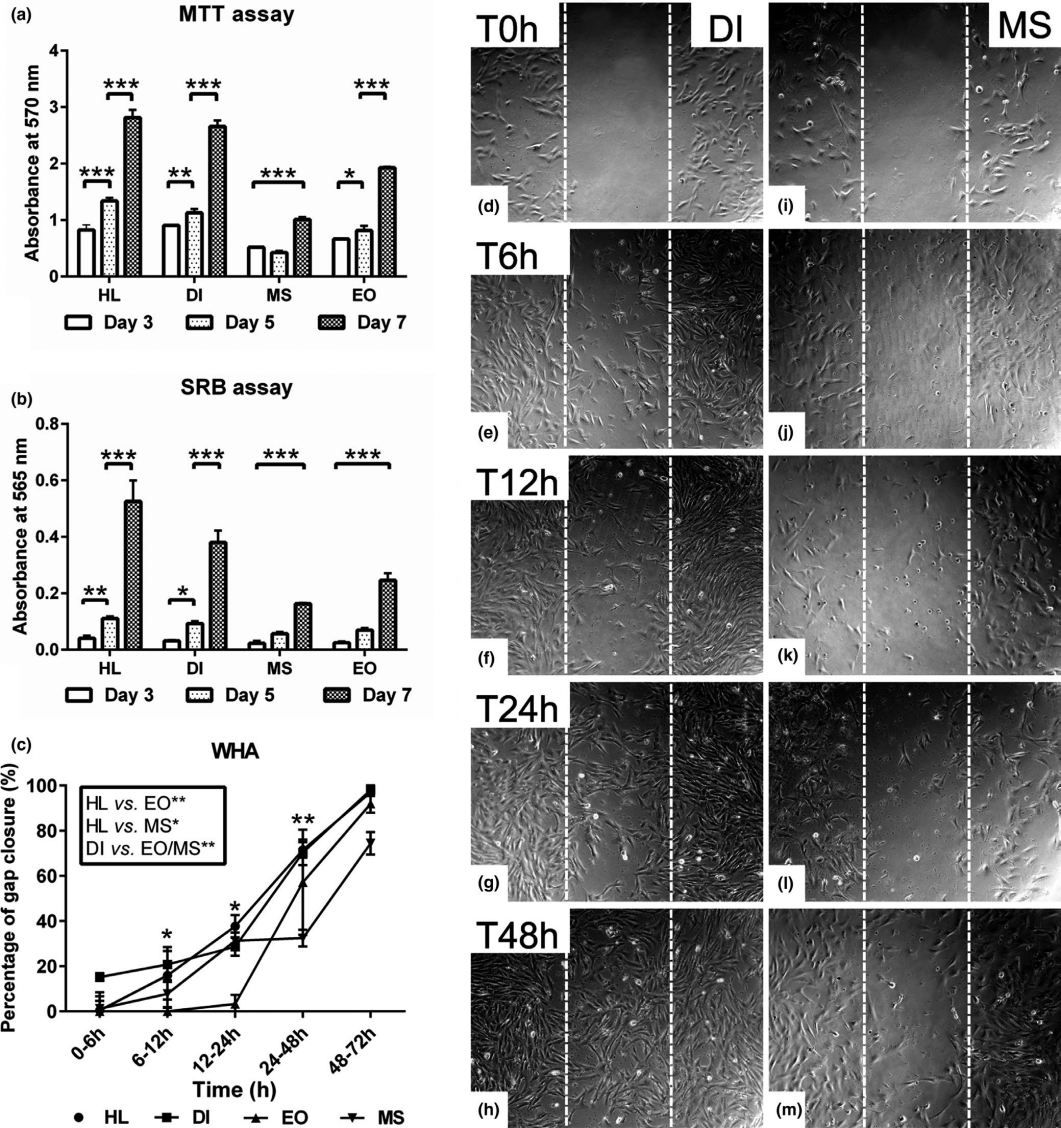
Evaluation of proliferation and migration of MSCs from various sources. (a, b) MSCs of ovine hind limb (HL), diaphragm (DI), extraocular (EO) and masseter (MS) muscles (*n* = 6) were cultivated in triplicate 5 × 10^3^ cells/well in growth medium for 3, 5 and 7 days. (a) MTT assay shows cell vibility for all experimental groups at different time points. The absorbance was measured at 570 nm. (b) Sulforhodamine B (SRB) assay for total cellular proteins reveals the average cell number in a timewise comparison. The absorbance was measured at 565 nm. (c) Wound‐healing assay (WHA) for HL, DI, EO and MS muscle cells in growth medium using silicon culture inserts. The analysis shows the average percentage of gap closure (area between the disrupted white lines) by migrating cells for up to 72 h. (d–m) Representative phase contrast images show enhanced cell migration in DI (d–h) compared to MS (i–m) muscle cells after 6, 12, 24 and 48 h in comparison to T0. All data are presented as mean ± SEM. *p < 0.05, **p < 0.01 and ***p < 0.001. Scale bare =200 μm

Cell viability was measured using the MTT colorimetric assay. Our data revealed increased cell viability in HL and DI cells (*p* < 0.001) compared to EO and MS muscle cells. A gradual increase in cell viability at day 7 in comparison to days 3 and 5 in the growth medium was detected in all experimental groups. However, MS cells displayed no alteration in cell viability up to day 5, followed by a significant increase on day 7. At all time points, HL and DI MSCs demonstrated higher viability compared to MS and EO muscle cells. There was no significant difference in cell viability between HL and DI MSCs on day 3. Similar results were obtained when comparing the MS and EO cells (Figure 1a). To examine cell cycle properties in all experimental groups, immunohistochemical staining for Ki67, a potent cell proliferation marker, was performed. The data showed marked increases in Ki67 nuclear antigen‐positive cells in HL and DI compared to the moderate and weak staining in EO and MS cells, respectively (Figure S2f,g,h,i).

The SRB assay assesses the total cellular protein content, indicative of the cell number. HL MSC population demonstrated a general increase compared to the DI, EO, and MS (*p* < 0.001) cells. Similar results were obtained by comparing DI cells with EO and MS (*p* < 0.01, *p* < 0.001) cells. However, no significant differences were found among the EO and MS muscle cells. Although, HL and DI cells showed an increase in number on days 5 (*p* < 0.01, *p* < 0.05) and 7 (*p* < 0.001) in comparison to day 3, MS and EO cells were increased in number on day 7 (*p* < 0.001) in comparison to previous time points. Conversely, the number of MS and EO MSCs showed no significant difference on days 3 and 5 (Figure 1b).

MSC migration capacity was assessed using wound‐healing assay. Cell migration to close the interface gap between the separated cell populations was determined by using culture inserts. Although the HL and DI MSCs displayed no significant difference in migration capacity, the HL cells demonstrated enhanced gap closure compared to EO (*p* < 0.01) and MS (*p* < 0.05) cells. A similar observation was made with the DI cells, which showed enhanced gap closure (*p* < 0.01) in comparison to the EO and MS muscle cells. DI and HL cells showed faster gap closure (*p* < 0.05) compared to EO and MS cells after 12 and 48 h, respectively. Data also indicated no significant change up to 6 h and after 48 h for all experimental groups (Figure 1c–m).

### 3.2 Characterisation of stemness for MSCs isolated from various muscle groups

**FIGURE 2:**
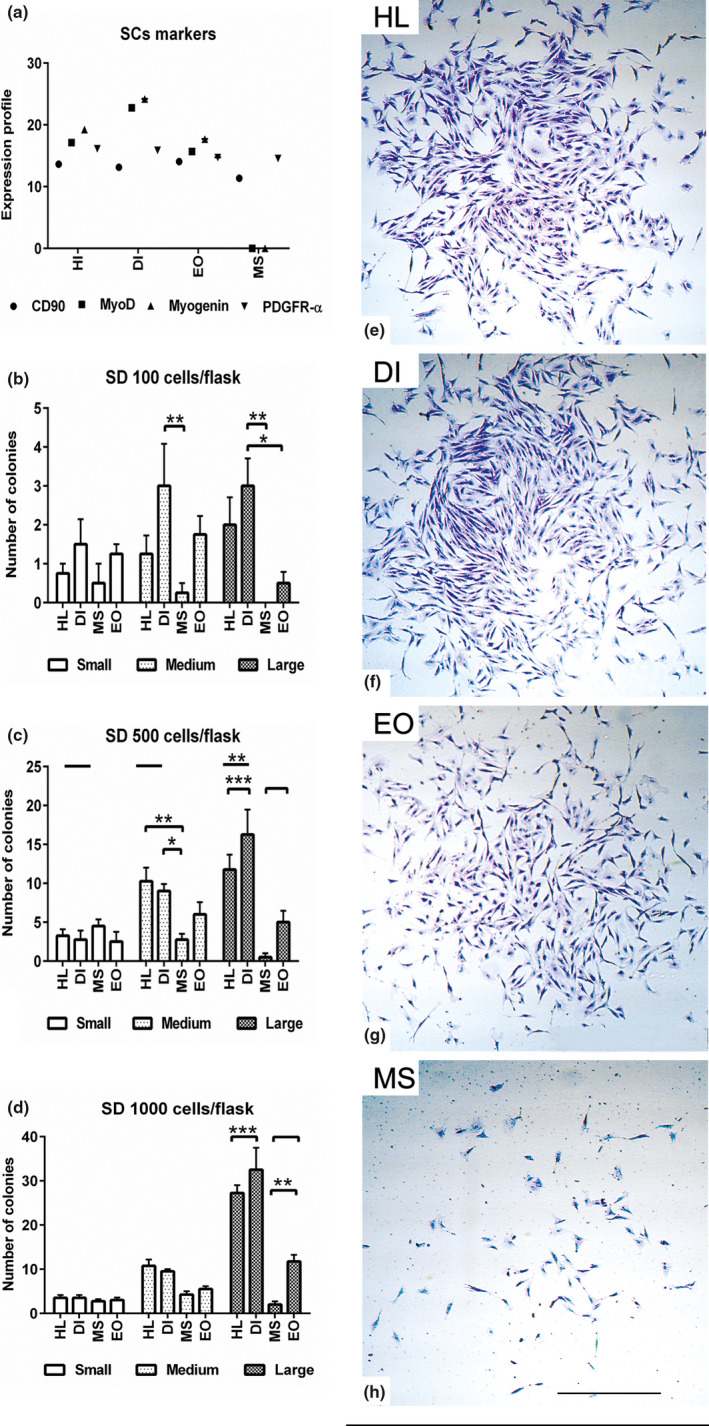
Characterisation of stemness for MSCs isolated from various muscle groups. (a) The expression profile of mesenchymal stem cells markers including *CD90*, *PDGFRα*, *MyoD* and *Myogenin* using PCR. Lysates of the HL, DI, EO and MS muscle cells were processed for RNA extraction and cDNA synthesis using a reverse transcription kit. (b–d) Colony forming assay evaluates the ability of stem cells to form colonies. HL, DI, EO and MS muscle cells were seeded at various seeding densities (SD), SD 1 × 10^2^ (b), 5 × 10^2^ (c) and 1 × 10^3^/culture flask. The number of small (10–20 cells), medium (21–50 cells) and large (50> cells) colonies were counted using crystal violet staining after 48 h. The analysis shows increased ability of HL and DI muscle cells to form medium and large colonies compared to EO and MS muscle cells. (e, f, g, h) Representative images show the comparison of colony morphology in HL, DI, EO and MS isolated muscle cells. All data presented as mean ± SEM. *p < 0.05, **p < 0.01 and ***p < 0.001. Scale bar =2 mm

Expression of stemness and myogenic markers was evaluated using immunocytochemistry, PCR, and flow cytometry after 48 h in growth medium. The isolated cells were immunopositive for Pax7, Myogenin, CD90, and α7‐integrin. Immunostaining for Myogenin was more obvious in HL, DI, and EO muscle cells than in MS muscle cells (Figure S1a–y). Analysis of flow cytometry data revealed approximately 99% immunoaffinity for CD44 and 0% for CD45 and MHCII in all muscle groups (Figure S1z). Interestingly, expression of the quiescent MSC marker, *Pax7*, was not detected using PCR in cells from any group. Alternatively, counting the number of Pax7‐positive cells from a minimum of ten microscopic fields per muscle showed that the HL muscle cells exhibited an increase in the number of Pax7‐positive cells compared to DI (*p* < 0.05), as well as EO and MS muscles (*p* < 0.001). Similarly, DI cells displayed a higher number of Pax7‐positive cells (*p* < 0.05) compared to the isolated EO and MS muscle cells (Figure S2a–d,k). PCR data revealed upregulated *MyoD* and *Myogenin* expression in the HL, DI, and EO muscle cells, but not in the MS muscle cells. Although all types of cells expressed *CD90*, the highest expression was found in MS muscle cells. Furthermore, upregulation of *PDGFRα* expression was observed in all cell types (Figure 2a).

The ability of MSCs to form colonies is an important criterion for evaluating stem cell properties. A CFU assay using different seeding densities was carried out. By using 1 × 10^2^ cells/25 cm^2^, a significant difference was detected by comparing the colony‐forming ability of MSCs (*p* < 0.001) between different cell groups. Although there was no significant difference between any of the groups in the formation of small colonies, DI MSCs displayed more medium colonies (*p* < 0.01) compared with the MS muscle cells. Similarly, DI muscle cells showed an increased number of large colonies compared with EO (*p* < 0.05) and MS (*p* < 0.01) muscle cells. MS MSCs displayed a weak ability to form large colonies at this seeding density (Figure 2b). At a seeding density of 5 × 10^2^ cells/25 cm^2^, HL and DI MSCs showed an increase in the number of medium (*p* < 0.01, *p* < 0.05) and large (*p* < 0.001) colonies compared to EO and MS muscle cells. Furthermore, DI muscle cells showed a higher ability to form large colonies (*p* < 0.001) compared to the HL cells (Figure 2c). At a seeding density of 1 × 10^3^/25 cm^2^, HL and DI cells showed a superior ability to form large colonies (*p* < 0.001) in comparison to the EO and MS muscle cells. Furthermore, EO muscle cells were able to form larger colonies (*p* < 0.01) in comparison to MS cells at high seeding density (Figure 2d,e–h).

### 3.3 Evaluation of MD in different MSC populations

**FIGURE 3:**
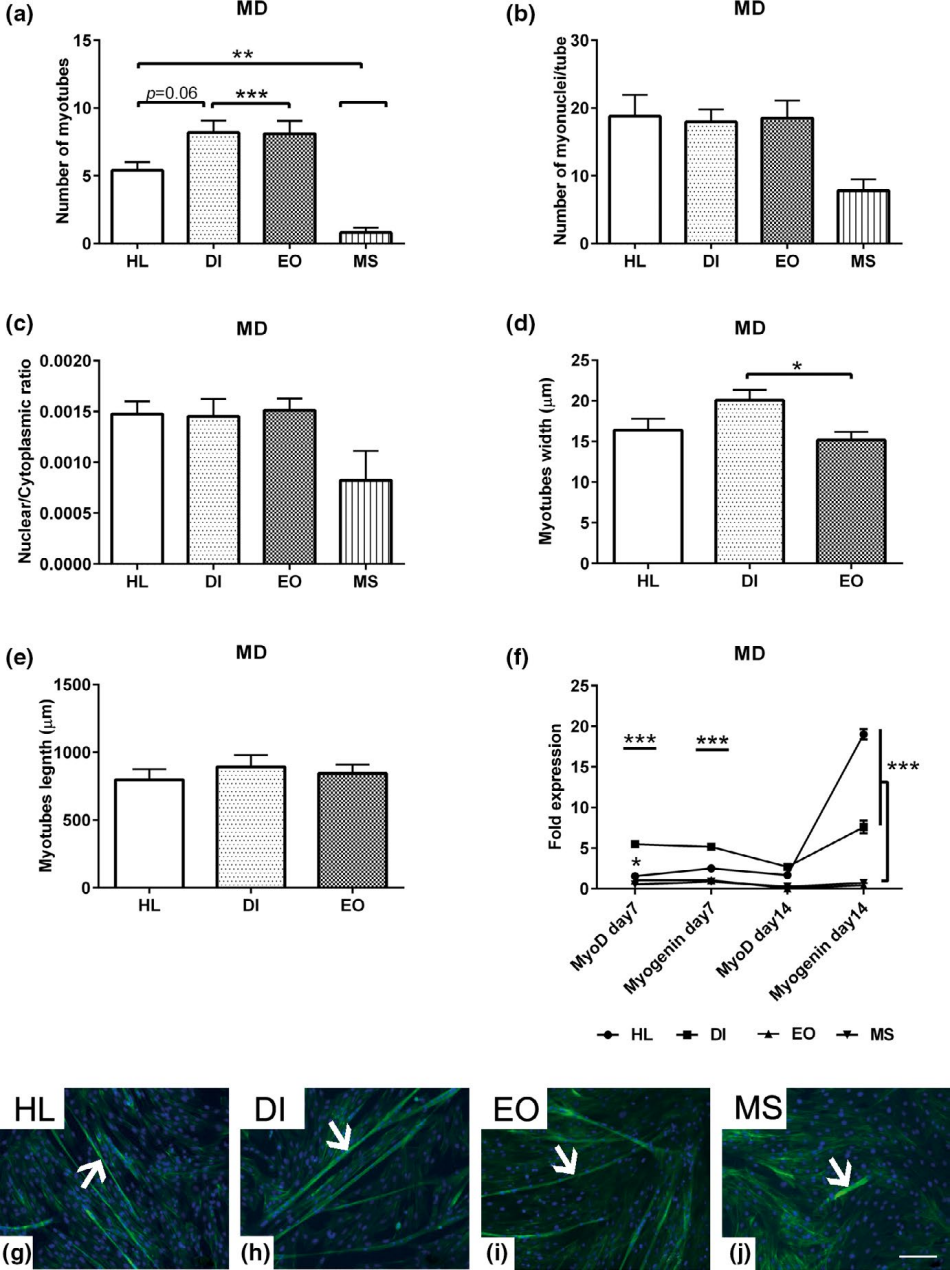
Evaluation of MD in different MSC populations. The cells of HL, DI, EO and MS muscles were seeded 1 × 10^4^ cells/well in growth medium for 48 h then were induced to MD up to day 14. (a) Morphometric analysis shows the number of myotubes per microscopic field (*n* = 10). The morphology of the myotubes were recognized using phalloidin staining (green). (b) Number of nuclei per tube (*n* = 17). (c) Measurement of the nuclear/cytoplasmic ratio (*n* = 10) for the myotubes. The total number of myonuclei was divided by the area of the myotube in order to calculate the nuclear/cytoplasmic ratio. (d) Analyses of the myotube width (*n* = 20) and (e) myotubes length show increased myotubes width in DI compared to EO muscle cells. (f) Fold expression of the MD markers; *MyoD* and *Myogenin* at day 7 and day 14 post induction. The expression fold was normalised to the non‐induced cells cultivated in basal medium using the equation of the 2‐^∆∆CT^. 18S1 house‐keeping genes were used as endogenous references. (g, h, I, j) Representative images for myotubes (white arrows) stained with phalloidin (green) show increased myogenic differentiation ability of HL and DI compared to EO and MS muscle cells. Cell nuclei were counterstained with DAPI (blue). All data presented as mean ±SEM. *p < 0.05, **p < 0.01 and ***p < 0.001. Scale bare =100 μm

The morphology of MSCs was examined on day 7 of MD using phalloidin staining. Evidence of MD via the formation of myotubes was observed in cells isolated from the HL, DI, and EO muscles. In contrast, MS MSCs displayed a weaker differentiation ability compared to the other cells (Figure 3g–j). For detailed comparison of MD capacity of MSCs derived from various muscle groups, morphometric analysis of myotubes was performed on day 7 of myogenic induction. The analysis showed an increased number of myotubes per microscopic field (*n* = 10) in the DI, EO (*p* < 0.001) and HL (*p* < 0.01) muscle cells compared to the MS muscle cells (Figure 3a). Neither the number of myonuclei per myotube, which indicates the rate of cell fusion, nor the nuclear/cytoplasmic ratio showed differences in any of the experimental groups (Figure 3b,c). To examine whether the MSC population affected the morphology of myotubes, the length and width (µm) of individual tubes (*n* = 10) were measured. Although there were no differences in the length of the tube in any of the groups, DI muscle cells differentiated into thicker tubes (*p* < 0.05) compared to the EO muscle cells. MS muscle cells were excluded from this analysis due to insufficient myotube formation for carrying out statistical analyses (Figure 3d,e). Expression of myogenic markers relative to the non‐induced control cells in BM was determined using RT‐qPCR. The analysis revealed upregulated *MyoD* expression in both DI (*p* < 0.001) and HL (*p* < 0.05) cells compared to the EO and MS muscle cells at day 7. A similar upregulation of *Myogenin* expression was detected in DI and HL cells (*p* < 0.001) at day 7 in comparison to the EO and MS muscle cells. The analysis revealed no significant differences in the levels of *MyoD* and *Myogenin* expression at day 7. Although the expression of *MyoD* in DI and HL muscle cells was downregulated at day 14, *Myogenin* expression showed a three‐fold upregulation in DI and twofold upregulation in HL muscle cells (*p* < 0.001) in comparison to EO and MS muscle cells. In contrast, MS cells displayed weak *MyoD* expression and later MD (Figure 3f).

### 3.4 Assessment of multipotency in the MSCs from different muscle sources

#### 3.4.1 Adipogenic differentiation

**FIGURE 4:**
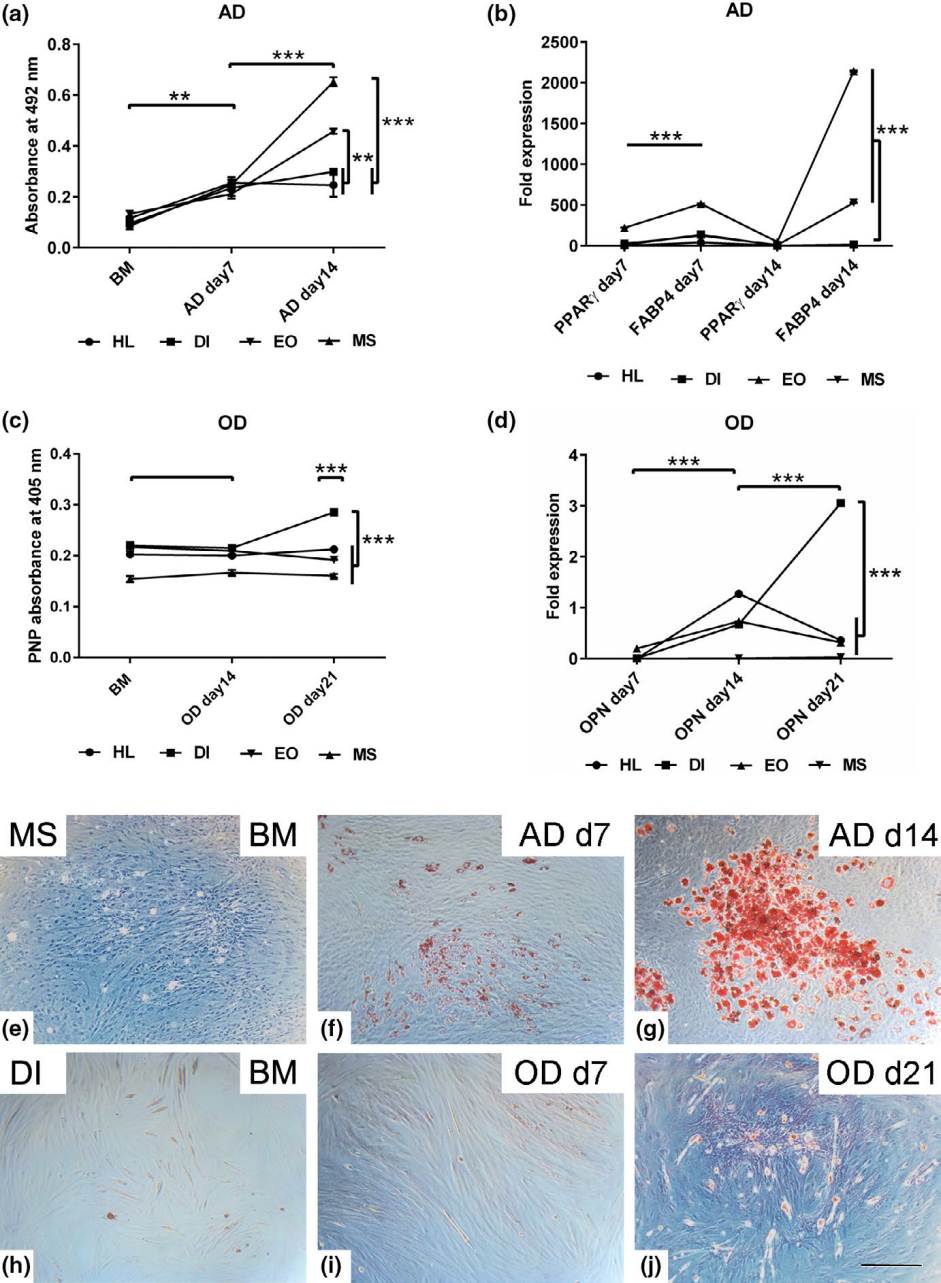
Assessment of multipotency in the MSCs from different muscle sources. MSCs of hind limb (HL), diaphragm (DI), extraocular (EO) and masseter (MS) muscles were cultivated 1 × 10^4^ cells/well in growth medium for 48 h then were induced into AD and OD for up to 21 days. (a) Semi‐quantification of Oil Red O staining at 492 nm absorbance shows an increased fat vacuole accumulation in MS and EO compared to HL and DI muscle cells. Non‐induced cells cultivated in basal medium (BM) served as a negative controls. (b) RT‐qPCR demonstrates fold increases of the adipogenic markers; *FABP4* and *PPARγ* expression at day7 and day14 post AD relative to non‐induced cells in BM. (c) Semi‐quantification of alkaline phosphatase (ALP) activity following OD induction shows promoted ALP activity in DI muscle cells at day 21. The p‐Nitrophenylphosphate is metabolized into p‐Nitrophenol (PNP) in the presence of ALP activity. The PNP absorbance was measured at 405 nm. (d) RT‐qPCR shows fold increase of *OPN* expression at day 14 and day 21 post OD in DI muscle cells. The expression fold was normalised to the non‐induced cells cultivated in basal medium using the equation of the 2‐^∆∆CT^. (e, f, g) Representative images of MS muscle cells after AD on day 7 and day 14, stained with Oil Red O, show fat vacuoles compared to BM muscle cells. (h, I, j) Images of DI cells stained with Alzarin Red S reveal calcium ions deposition (red) following OD induction at day 21 compared to cells in BM and day 7 of OD. All data are presented as mean ± SEM. **p < 0.01 and ***p < 0.001. Scale bare =200 μm

Adipogenic differentiation was examined on days 7 and 14 after induction. Oil Red O staining demonstrated the formation of fat vacuoles in all induced cells compared to the non‐induced cells. MS and EO cells displayed a marked increase in fat vacuole accumulation compared to the HL and DI muscle cells (Figure 4e–g). Semi‐quantification of Oil Red O staining on day 7 showed higher fat content in the MS, HL, and DI muscle cells (*p* < 0.01) compared to the cells cultivated in BM. Similar results were detected in all experimental groups at day 14 post‐adipogenic induction (*p* < 0.001) compared to the cells in BM. The analysis demonstrated enhanced fat formation in MS and EO cells at day 14 compared to the cells of the same muscles at day 7 (*p* < 0.001), as well as in comparison to HL and DI muscle cells at day 14 (*p* < 0.001, *p* < 0.01, respectively) (Figure 4a,e–g). Quantification of the adipogenic markers revealed upregulated *PPARγ* and *FABP4* expression on day 7 in all experimental groups compared to the corresponding cells in BM. However, EO muscle cells demonstrated an even higher fold increase (*p* < 0.001) compared to the other cell types at day 7. Although the level of *PPARγ* was downregulated in all induced cells at day 14, the expression of *FABP4* was persistent in the induced EO and MS cells (*p* < 0.001) compared to that in the HL and DI muscle cells (Figure 4b).

#### 3.4.2 Osteogenic differentiation

Alizarin Red S staining was performed on days 7, 14, and 21 to evaluate calcium ions deposition indicative of OD. Although no staining was observed up to day 14 post‐OD induction, small, red‐stained nodules were detected in the DI muscle cells at day 21 post‐induction (Figure 4h–j). In contrast, MS cells showed no positive staining during the induction process. Furthermore, ALP activity was measured at days 14 and 21 after OD induction. The analysis revealed an increased ALP activity in DI (*p* < 0.001) and EO (*p* < 0.05, *p* < 0.01) muscle cells at day 21 in comparison to both osteogenically induced cells at day 14 and non‐induced cells cultivated in BM. The elevated ALP activity in DI muscle cells on day 21 was comparatively higher (*p* < 0.001) in all experimental groups at the same time point (Figure 4c). No significant difference in ALP activity was detected between the induced and non‐induced MS muscle cells. At the molecular level, the expression of *OPN* in OD‐induced cells was quantified on days 7, 14, and 21 using RT‐qPCR. Although the analysis showed no *OPN* expression at day 7 in any of the induced cells, its expression was upregulated on day 14 in the HL, DI, and EO cells (*p* < 0.001) compared to the control non‐induced cells in BM. Although *OPN* expression was downregulated on day 21 in DI, EO, and MS induced cells (*p* < 0.001) compared to day 14, HL muscle cells showed a twofold increase (*p* < 0.001) in *OPN* expression at the same time point (Figure 4d).

## 4 DISCUSSION

Skeletal MSCs provide an interesting tool not only for understanding MD, but also for investigating tissue regeneration from a therapeutic perspective. We hypothesised that muscle origin regulates their myogenic fate and reflects their regeneration capacity. Thus, we investigated the stemness, MD potential, and multipotency of ovine MSCs from various embryonic sources. All experimental groups revealed expression of MSCs and myogenic markers, as shown using immunohistochemistry, PCR, and flow cytometry. Although Pax7 antigen was clearly observed and quantified using immunostaining, the mRNA expression of *Pax7* was undetectable even in early passages (P2–P4). A possible explanation is that *Pax7* was rapidly downregulated to an undetectable level or that mRNA expression was rapidly turned over in the activated muscle precursors. It was found that Pax7 is the most reliable marker for MSC identification (Kuang & Rudnicki, 2008; McCullagh & Perlingeiro, 2015). Some studies have reported that Pax7 is a marker of quiescent satellite cells (Kuang & Rudnicki, 2008; Shioi et al., 1995), and its expression decreases shortly after MSC isolation and passaging (Ding et al., 2017). Similarly, it has been documented that after muscle injury, MSCs become activated and express *MyoD* and *Myogenin* upon commitment to MD (Zammit et al., 2004, 2006). The data revealed strong expression of *PDGFRα* in MSCs, which is in agreement with a previous report suggesting its expression in skeletal muscles by fibro‐adipogenic progenitors (Joe et al., 2010). Furthermore, the latter might play a positive role in muscle regeneration by producing a scaffold for efficient myogenesis as previously reported (Watt et al., 1987).

The adherence and colony‐forming ability of MSCs have been considered as one of the characteristic features of stem cells. It has been reported that stem cells that efficiently form colonies are more proliferative and possess a greater capacity to differentiate (Pochampally, 2008). The data revealed that DI and HL muscle cells had higher colony‐forming capacity, viability, and proliferation rate compared to EO and MS muscle cells. Thus, our data suggest an enhanced regenerative ability for the trunk muscles compared to head muscles, which probably requires a higher cell dose to perform in a similar manner when considering cell transplantation. The wound‐healing assay, which is an indicative tool for cell migration, demonstrated that the HL and DI muscle cells have a superior potential for cell migration compared to cells of the head mesoderm, including the EO and MS muscles. Previous studies have shown that MSCs possess extensive cell mobility between myofibers and adjacent muscles across the extracellular matrix during muscle development and regeneration (Hughes & Blau, 1990; Westerblad et al., 2010). By considering the difference in the embryonic origin of the muscles from which the cells were isolated as HL and DI muscles originate from somites, whereas, MS and EO muscle cells are derived from the head mesoderm (Harel et al., 2009; Zammit et al., 2004), suggesting that somite cells have conserved stem cell properties compared to the cells of the head mesoderm. Moreover, the superior regenerative capacity of cells from trunk muscles suggests that head muscles utilise different myogenic programmes to achieve muscle repair. It has been reported that head muscles follow a specialised programme to produce satellite cells for initiating MD and post‐natal muscle regeneration (Nogueira et al., 2015). Similarly, a study showed that eye muscles are not affected in some muscular dystrophies causing weakness and degeneration of body musculature (Emery, 2002).

In the present study, HL and DI muscle cells showed efficient MD, as indicated by upregulated expression of *MyoD* at day 7 and *Myogenin* at day 14 and abundant myotube formation. In agreement with our results, a study demonstrated that DI MSCs exhibit a significant rate of myogenesis compared to cells from the semimembranosus muscle of the HL (Redshaw & Loughna, 2012; Redshaw et al., 2010). Interestingly, the DI muscle cells showed wider myotubes compared to the EO muscle cells, which might be due to the future differences between both muscles, as fine and rapidly contractile fibres are required for eye movement (Zhou et al., 2011). The data highlighted that impaired MD in MS cells might be related to either a poor proliferation rate or a different myogenic programme orchestrating the development of the head muscles. In this context, it has been reported that the regulatory pathways that control skeletal myogenesis and cell fate are different in trunk muscles compared to the muscles of the head region (Harel et al., 2009). Similarly, it has been found that several genes are involved in the specification of pre‐myogenic progenitors in the trunk, including *Pax3* and ladybird homeobox1 (*Lbx1*); however, other molecular cues control head muscle specification. For example, the bicoid‐related homeobox transcription factor (*Pitx2*) is required for head myogenesis, because depletion of *Pitx2* causes complete absence of EO and masticatory muscle development (Shih et al., 2007, 2008).

The cells of MS and EO muscles possessed multipotency toward the adipogenic lineage, as shown by Oil Red O staining for fat vacuole formation and upregulation of *FABP4* and *PPARγ* expression. Interestingly, HL and DI muscle cells displayed moderate adipogenic potential and were more prone to MD. Our study did not reveal any difference in lipid accumulation between HL and DI muscle cells. In contrast, another study showed that semimembranosus muscle cells had greater lipid accumulation compared to the DI (Redshaw & Loughna, 2012). It was reported that *FABP4* is normally expressed in skeletal muscle fibres, and its expression is increased during exercise (Fischer et al., 2006). The latter could be due to enhanced metabolic activity and oxidative capacity during exercise rather than a greater adipogenic potential.

Evaluation of OD revealed enhanced ALP activity, a common biochemical marker for osteoblast activity (Sabourin & Rudnicki, 2000), calcium ions deposition at day 21, and upregulated *OPN* expression in the HL and DI muscle cells. Upregulated *OPN* expression is an indicative marker for osteogenic commitment; *OPN* expression was observed in the course of OD of mesenchymal stem cells, which suggests its important role in osteogenesis (Zohar et al., 1998). *OPN* is not only expressed in osteoblasts, but also biosynthesised by myoblasts (Uezumi et al., 2010). *OPN* has been reported to play a role in skeletal muscle inflammation and fibrosis following injury (Pagel et al., 2014). A similar report revealed that *OPN* is produced in muscle cells as well as macrophages as an acute response after injury to orchestrate muscle repair (Wasgewatte Wijesinghe et al., 2019). In contrast, MS muscle cells showed impaired osteogenic capacity, probably due to either limited multipotency or the tendency toward AD.

Our data revealed that the myogenic programme of the trunk muscles, including HL and DI, was comparatively different from the head muscles, not only from the embryonic perspective, but also considering their regenerative potential. A large body of evidence has highlighted the differences in structural components between the trunk and head muscles. A recent study demonstrated that adult human beings EO and MS muscles possess novel myosins, unusual fibre morphology, and a combination of different myosin isoforms of unknown functional significance (Seale et al., 2000). Similar studies have indicated that EO and MS muscles express more developmental myosin isoforms that disappear in other adult muscles (D'Albis et al., 1986; Will et al., 2015). Several other factors considered to modulate the pattern of MSCs, such as breed, gender, post‐natal age, muscle fibre origin, and phenotype, have an effect on MSC number, proliferation rate, and transcription factor expression *in vitro* (Harding et al., 2015; Manzano et al., 2011; Torrente et al., 2004). In the same context, the number of MSCs varies between different muscles and within the same muscle between different fibre types (Ding et al., 2017; Gibson & Schultz, 1982; Keefe et al., 2015; Zammit et al., 2004). For example, DI muscles displayed more MSCs per unit volume than the limb muscles (Keefe et al., 2015). Furthermore, maternal nutrition during gestation may influence the properties of muscle cells. A study demonstrated that restricted maternal nutrition during gestation alters the expression of myogenic regulatory factors, changes the proliferation rate, and impairs MD in MSCs of lambs (Raja et al., 2016). Moreover, the number of foetuses during gestation may be a determinant factor for post‐natal muscle performance. A study has revealed that twin foetuses/neonates showed less muscle weight compared to singles due to reduced myofiber cross‐sectional area and lower number of myogenic progenitors. The latter might play a role in myofiber hypertrophy in late gestation as well as early post‐natal period (McCoard et al., 2001). Alternatively, maternal obesity in sheep may enhance intramuscular adipogenesis during foetal development (Yin et al., 2013) and possibly predispose MSCs to adipogenic induction.

## 5 CONCLUSION

MSCs offer a promising tool for regenerative medicine and cell‐based therapy. Evaluation of stem cell populations would improve our understanding of the regenerative ability of each muscle. We show that ovine MSCs isolated from different muscle groups express specific stem cell and myogenic markers. By comparing the colony formation potential, viability, proliferation, and migration capacity, we concluded that the cells of the HL and DI muscles possess a greater regenerative potential compared to those of EO and MS muscles. Head muscle cells had a higher adipogenic capacity compared to the trunk muscle cells. In contrast, cells of trunk muscles displayed enhanced osteogenic potential, as shown by increased ALP activity and upregulated *OPN* expression. Collectively, these results suggest that MSCs derived from the paraxial mesoderm and head mesoderm followed a different myogenic programme. Additionally, we could assume that the DI and HL muscle cells are more suitable sources for biopsies and stem cell isolation for tissue regeneration.

## CONFLICT OF INTEREST

The authors have declared no conflict of interest.

## AUTHOR CONTRIBUTIONS

MIE, KG, AE, MCK collected the raw data set and analyzed the results. MIE wrote the original draft of the manuscript and interpreted the results, SW and SA revised and finalized the submitted version of the manuscript.

## Supporting information

Figure S1.

Figure S2.

## Data Availability

The data collected and the analyses performed to generate the manuscript results are available from the corresponding author on reasonable request.
