# Supplementary figures and images for: Characterisation of stemness and multipotency of ovine muscle‐derived stem cells from various muscle sources
Source: J Anat. 2021 Feb 27;239(2):336–50. doi: 10.1111/joa.13420 (PMC8273587; doi:10.1111/joa.13420)

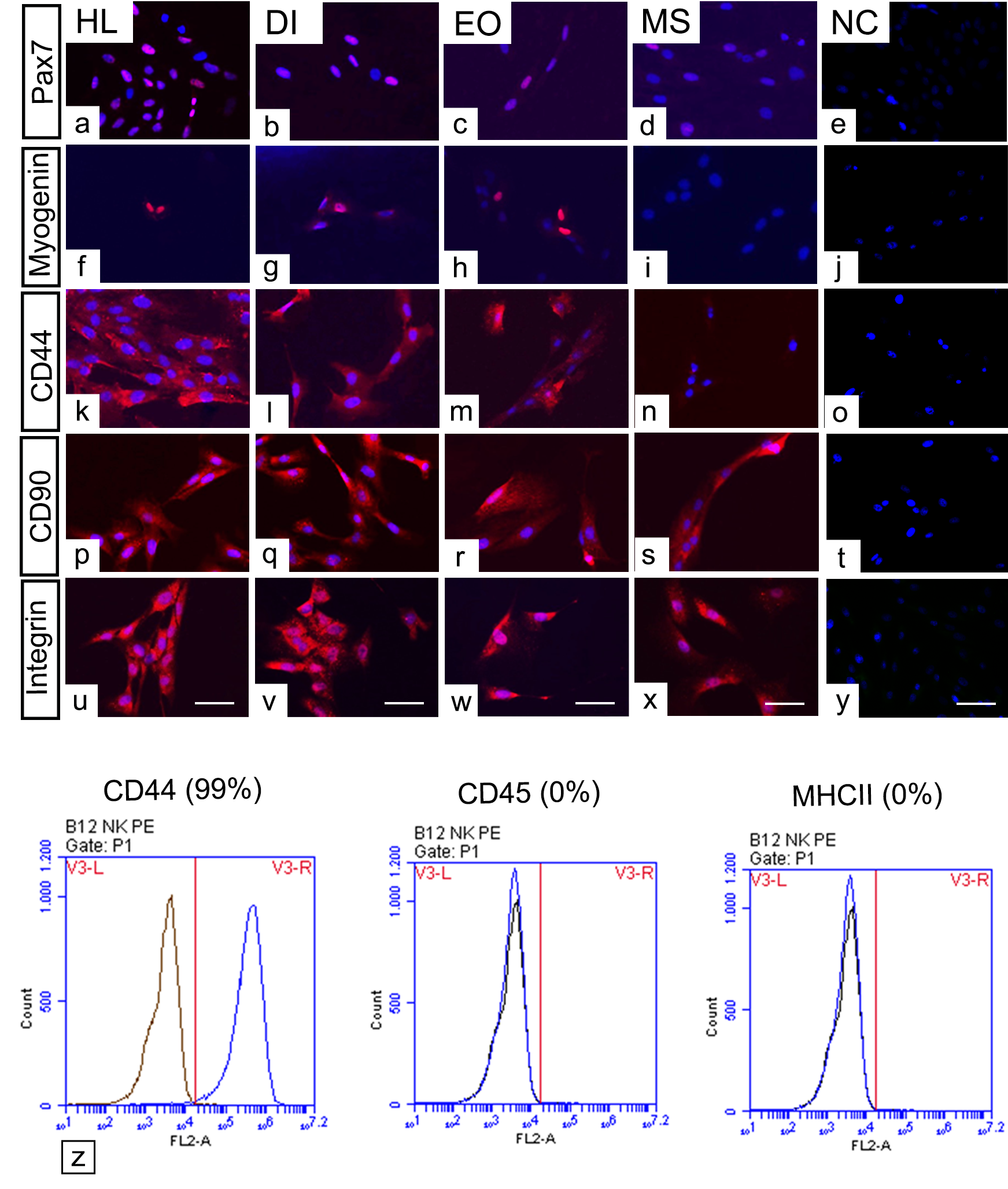

Supplement: Supplementary file 1 — Figure S1. [file JOA-239-336-s002.tif]

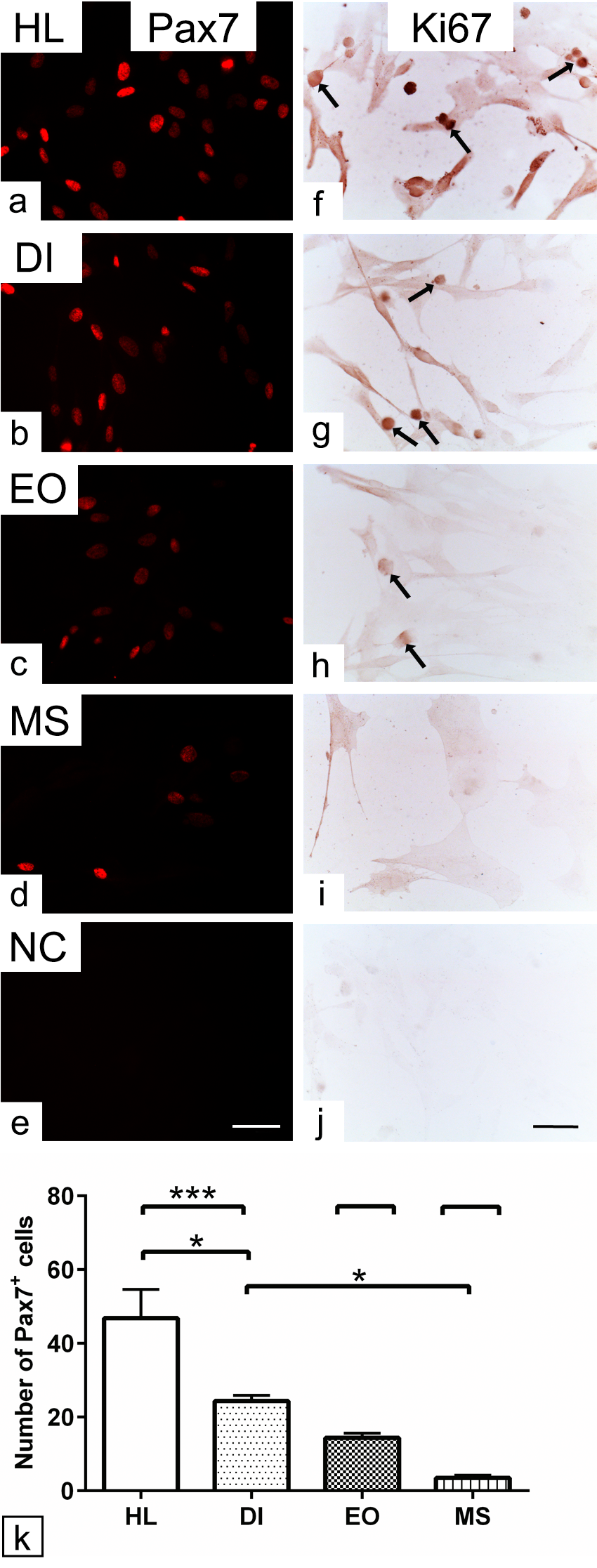

Supplement: Supplementary file 2 — Figure S2. [file JOA-239-336-s001.tif]
